# Association of smoking status, cumulative smoking, duration of smoking cessation, age of starting smoking, and depression in Korean adults

**DOI:** 10.1186/1471-2458-12-724

**Published:** 2012-08-31

**Authors:** Woo-Jun Yun, Min-Ho Shin, Sun-Seog Kweon, So-Yeon Ryu, Jung-Ae Rhee

**Affiliations:** 1 Department of Preventive Medicine, Chonnam National University Medical School, Hak-1-dong, Dong-gu, Gwangju, 501-746, Republic of Korea; 2 Jeonnam Regional Cancer Center, Chonnam National University Hwasun Hospital, Jeollanamdo, 519-809, Republic of Korea; 3 Department of Preventive Medicine, Chosun University Medical School, Gwangju, 501-759, Republic of Korea

## Abstract

**Background:**

Many previous studies did not sufficiently control for several confounding factors that may affect the association between smoking and depression, such as socioeconomic status. We investigated the association between depression and smoking status, smoking exposure, duration of smoking cessation, and age of starting smoking while controlling for socioeconomic factors.

**Methods:**

This study was based on a community health survey performed in Jeollanam-do, South Korea, between September and November 2009. In total, 20,084 subjects (9,118 males and 10,966 females) were included in the analysis. Information on smoking characteristics, such as smoking status, pack-years of smoking, and age of starting smoking, was collected using a standardized questionnaire. Depression was defined using the Korean CES-D score.

**Results:**

The odds ratios (ORs) of depression were 1.35 (0.92–1.98) for former smokers and 1.77 (1.27–2.48) for current-smokers among males, and 2.67 (1.38–5.16) for former smokers and 3.72 (2.11–6.54) for current-smokers among females, after adjusting for other confounding factors. Compared to light smoking, heavy smoking was significantly associated with depression in males [OR = 3.97, 95% confidence interval (CI) = 1.42–11.14], but not in females (OR = 1.24, 95% CI = 0.73–2.09). No significant associations between depression and age of starting smoking and duration of smoking cessation were observed among former smokers.

**Conclusions:**

Our data demonstrate that smoking is strongly associated with depression, particularly among females. These findings suggest that depression prevention may need to be combined with smoking prevention and that different strategies may be needed for males and females.

## Background

Epidemiologic [1-4] and clinical [5,6] studies have reported a strong association between smoking and depression. Several explanations for this association have been proposed.

The first is that underlying environmental or genetic factors predispose individuals to both smoking and depression [2,7-9]. The second is that depression leads to smoking. Several studies reported that smoking may increase as a result of self-medication for depression [10-12]. The third is that smoking itself increases the risk of depression [13-18]. Some studies found that smoking among adolescents increased the risk of depression, and another reported that smoking was highly associated with depression in adults. However, in these studies, many confounding factors, such as socioeconomic status, which may affect the association between smoking and depression, were not sufficiently controlled. Furthermore, to our knowledge, no previous study has simultaneously evaluated several aspects of smoking status, including the amount of smoking, age of starting smoking, and duration of smoking cessation.

The objective of this study was to investigate the cross-sectional association between smoking and depression after controlling for socioeconomic factors and many aspects of smoking in population-based subjects.

## Methods

### Subjects

This study was based on a community health survey performed in Jeollanam-do, South Korea, between September and November 2009. This population-based cross-sectional survey was designed to investigate the health status, health behaviors, use of medical facilities, quality of life, and socioeconomic state of the community. A complex, multistage, probability sampling design was used to select participants. The study included 20,097 subjects aged 19–105 years. Thirteen subjects were excluded from the study because of missing smoking status information and the Center for Epidemiological Studies–Depression Scale (CES-D) score. Finally, the analysis included 20,084 subjects (9,118 males and 10,966 females). No institutional review board approval was required for this study because the data are available for public use and void of identifiers.

### Definition of depression

The CES-D is a short self-report scale designed to measure depression in the general population and is widely used for epidemiologic studies on depression. The scale asks about the existence of 20 symptoms during the last week; scores range from 0 to 60, with higher scores indicating greater depression [19].

Cho et al. translated the CES-D scale into Korean, paying attention to culturally different modes of expression of depressive feelings and thoughts. They reported that the Korean CES-D was reliable and valid for the Korean population, and reported that the appropriate cutoff point for depression was a Korean CES-D score of 21 [20]. Here, we defined depression as a Korean CES-D score ≥21.

### Definition of smoking

Smoking status was classified as never-smokers (smoked <100 cigarettes in their lifetime and not currently smoking), former smokers (smoked ≥100 cigarettes in their lifetime and currently a nonsmoker), and current-smokers (smoked ≥100 cigarettes in their lifetime and currently a smoker). Status of smoking exposure was classified as light smoker (≤10 cigarettes daily), moderate smoker (≤20 cigarettes daily), and heavy smoker (>20 cigarettes daily). Pack-years of smoking were calculated by multiplying the number of years smoked by the average number of packs per day. Based on the age of starting smoking, subjects were classified as ≤19 years, 20–24 years, and ≥25 years, and compared with never-smokers. Based on the duration of smoking, age of starting smoking, and age of former smokers, we calculated the duration of smoking cessation. Former-smokers were classified as <5 years, 5–9 years, 10–19 years, and ≥20 years, and compared with current-smokers.

### Covariates

Household income was determined by each subject’s reported monthly household income in Korean won and categorized as: < one million won; < two million won; < three million won; and ≥ three million won. Education status was categorized as uneducated, elementary and middle school, high school, and college. Marital status was coded into two categories (married/partnered, never married/non-partnered). Job status was coded into three categories (nonmanual, manual, others) according to the Korean Standard Classification of Occupation (KSCO).

Monthly drinking was defined as drinking alcohol at least once per month in the previous year. We used monthly drinking as drinking status (yes/no) in this study. Hypertension was defined as the use of medication for hypertension. Diabetes was defined as the use of medication for diabetes. Body mass index (BMI) was calculated using self-reported height and weight.

### Statistical analysis

All analysis in this study was performed separately for both sexes because of differences in smoking status and depression rate according to sex, as well as the interaction effects between sex and smoking status on depression. The characteristics of the study population are expressed as the mean ± standard error or number (weighted percentage). Multivariate survey logistic regression was used to evaluate the association of smoking characteristics (e.g., smoking status, cumulative smoking exposure, age of starting smoking, and duration of smoking cessation) with depression. The odds ratio (OR) and 95% confidence interval (CI) was calculated in unadjusted and multivariate-adjusted models after adjusting for age, income status, education status, marital status, job status, drinking status, hypertension, diabetes mellitus, cerebrovascular disease (CVD), BMI, and physical activity. The multivariate-adjusted model of smoking exposure among current-smokers was further adjusted for the amount of smoking, and the multivariate-adjusted model of the duration of smoking cessation among former-smokers was further adjusted for pack-years of smoking. The multivariate-adjusted model of age of starting smoking among current-smokers was additionally adjusted for th4e amount of smoking.

All statistical analyses were performed using STATA software, version 11.0 SE (STATA, College Station, TX, USA).

## Results

### Characteristics of the study population

Of the 20,084subjects analyzed, 9,118 (49.3%) were male and 10,966 (50.6%) female. The point prevalence of depression was 4.6% in males and 8.0% in females. Subjects with depression were significantly older than those without depression in both sexes (males: 46.5 ± 0.4 vs. 56.4 ± 2; females: 50.1 ± 0.5 vs. 58.6 ± 1.8). Subjects with depression had a significantly higher percentage of current/former smokers than subjects without depression in both sexes (males: 45.0% vs. 49.1%; females: 1.6% vs. 7.8%).

Subjects with depression had significantly higher mean pack-years of smoking than those without depression in both sexes (males: 13.8 ± 0.4 vs. 20.3 ± 2.4; females: 0.2 ± 0.2 vs. 1.3 ± 0.5). Subjects with depression did not exhibit a significantly lower mean age of starting smoking than those without depression in both sexes (males: 20.7 ± 0.1 vs. 20.8 ± 0.6; females: 31.0 ± 2.0 vs. 35.6 ± 5.3). Subjects with depression had a significantly longer duration of quitting smoking than those without depression in males, but not in females (males: 13.0 ± 0.6 vs. 17.9 ± 3.9; females: 16.3 ± 4.0 vs. 15.5 ± 6.5) (Table 1).

**Table 1:** General characteristics of the study population with and without depression (n = 20,084)

**Characteristics**	**Men (n = 9,118)**		**Women (n = 10,966)**	
	**Non-depression (n = 8,702)**	**Depression (n = 416)**	**Non-depression (n = 10,094)**	**Depression (n = 872)**
Age (years)	46.5 ± 0.4	56.4 ± 2.1*	50.1 ± 0.5	58.6 ± 1.8*
Smoking status				
Never smokers	2,687 (30.9)	99 (22.1)*	9,843 (97.7)	798 (89.3)^†^
Former smokers	2,333 (24.0)	138 (28.7)	76 (0.3)	23 (2.8)
Current smokers	3,682 (45.0)	179 (49.1)	175 (1.6)	51 (7.8)
Pack-years of smoking	13.8 ± 0.4	20.3 ± 2.4*	0.2 ± 0.2	1.3 ± 0.5
Age of starting smoking	20.7 ± 0.1	20.8 ± 0.6	31.0 ± 2.0	35.6 ± 5.3
Duration of smoking cessation	13.0 ± 0.6	17.9 ± 3.9*	16.3 ± 4.0	15.5 ± 6.5*
Household Income				
< one million won	2,494 (20.1)	260 (53.6)*	3,735 (26.7)	538 (53.2)*
< two million won	2,127 (21.1)	75 (18.3)	2,256 (20.6)	129 (15.9)
< three million won	1,655 (21.8)	27 (9.7)	1,647 (19.2)	94 (15.0)
≧ three million won	2,421 (36.8)	53 (18.2)	2,450 (33.3)	110 (15.7)
Education level				
Uneducated	805 (5.8)	116 (22.2)*	3,153 (22.1)	489 (48.3)*
Elementary and Middle school	3,360 (28.3)	176 (38.0)	3,497 (29.4)	209 (22.5)
High school and College	4,537 (65.8)	123 (39.7)	3,441 (48.4)	174 (29.0)
Marital status				
Married/partnered	6,573 (68.5)	255 (54.2)*	6,482 (64.4)	363 (40.2)*
Never married/non-partnered	2,129 (31.4)	160 (45.7)	3,611 (35.5)	509 (59.7)
Job status				
Non-manual	1,332 (19.5)	20 (6.8)*	982 (13.9)	43 (6.8)*
Manual	5,814 (61.2)	155 (37.1)	4,518 (36.9)	237 (25.1)
Others	1,558 (19.2)	241 (56.0)	4,594 (49.0)	592 (68.0)
Hypertension	1,607 (13.7)	118 (24.6)*	2,475 (18.3)	319 (33.8)*
Diabetes Mellitus	715 (6.5)	63 (14.5)^†^	766 (5.9)	119 (12.6)^†^
Cerebrovascular disease	363 (3.0)	65 (8.9)*	413 (3.2)	100 (9.8)*
Body Mass Index (BMI)	24.0 ± 0.2	24.2 ± 1.8	24.6 ± 0.4	28.1 ± 2.1*
Physical activity	2,971 (33.2)	82 (19.4)*	2,608 (23.8)	164 (16.8)*

Data are presented as mean (standard error) or unweighted number (weighted percent).

* p <0.001.

^†^ p <0.05.

### Smoking status and depression

We found interaction effects between sex and current smoking on depression in the survey logistic regression analyses (*p* = 0.009 for interaction).

Currents smokers had a significantly higher risk of depression than those who had never smoked in both males (OR = 1.77, 95% CI = 1.27–2.48) and females (OR = 3.72, 95% CI = 2.11–6.54). However, former smokers had a significantly higher risk of depression than those who had never smoked in females (OR = 2.67, 95% CI = 1.38–5.16), but not in males (Table 2).

**Table 2:** Odds ratio for depression according to smoking status

	**Smoking status**	**Smoking status**	
	**Never smokers (n = 13,427)**	**Former smokers (n = 2,570)**	**Current smokers (n = 4,087)**
Men	(n = 2,786)	(n = 2,471)	(n = 3,861)
Unadjusted	1.00 (Reference)	1.66 (1.18-2.35)	1.52 (1.11-2.09)
Multivariate- adjusted*	1.00 (Reference)	1.35 (0.92-1.98)	1.77 (1.27-2.48)
Women	(n = 10,641)	(n = 99)	(n = 226)
Unadjusted	1.00 (Reference)	4.68 (2.38-9.20)	5.30 (3.30-8.50)
Multivariate- adjusted*	1.00 (Reference)	2.67 (1.38-5.16)	3.72 (2.11-6.54)

Data are presented as mean (standard error) or odds ratio (95% confidence interval).

*Adjusted for age, income status, education status, marital status, job status, drinking status, BMI, physical activity, Hypertension, Diabetes mellitus, and Cerebrovascular disease.

Current smoking exposure had a significantly higher risk for depression among current-smokers in females, but not in males. Compared to light-smokers, heavy-smokers had a significantly higher risk of depression in females (OR = 3.97, 95% CI = 1.42–11.14). However, the OR of heavy-smokers for depression in males was 1.24 (95% CI = 0.73–2.09) (Table 3).

**Table 3:** Odds ratio for depression according to smoking exposure among current smokers

	**Cumulative smoking exposure**		
	**Light smokers [≦10.0] (n = 615)**	**Moderate smokers [10.1 – 20.0] (n = 1,259)**	**Heavy smokers [>20.0] (n = 2,213)**
Men	(n = 515)	(n = 1,181)	(n = 2,164)
Unadjusted	1.00 (Reference)	0.53 (0.28-0.98)	0.83 (0.47-1.45)
Multivariate- adjusted*	1.00 (Reference)	0.71 (0.40-1.26)	1.24 (0.73-2.09)
Women	(n = 100)	(n = 78)	(n = 49)
Unadjusted	1.00 (Reference)	0.58 (0.19-1.69)	3.65 (1.15-11.59)
Multivariate- adjusted*	1.00 (Reference)	0.63 (0.23-1.69)	3.97 (1.42-11.14)

Data are presented as mean (standard error) or odds ratio (95% confidence interval).

*Adjusted for age, income status, education status, marital status, job status, drinking status, BMI, physical activity, Hypertension, Diabetes mellitus, and Cerebrovascular disease.

Age of starting smoking did not have a significantly higher risk for depression among current-smokers. Compared to those who started smoking at ≤19 years, the ORs of those who started smoking at 20–24 years were 0.81 (95% CI = 0.52–1.27) in males and 2.18 (95% CI = 0.37–12.69) in females. ORs of those who started smoking at >20 years were 1.15 (95% CI = 0.61–2.17) in males and 0.95 (95% CI = 0.22–3.93) in females (Table 4).

**Table 4:** Odds ratio for depression according to age of starting smoking among current smokers

	**Age of starting smoking**		
	**< 19 years (n = 1,281)**	**20 - 24 years (n = 2,014)**	**≧ 25 years (n = 789)**
Men	(n = 1,259)	(n = 1,980)	(n = 618)
Unadjusted	1.00 (Reference)	0.79 (0.51-1.22)	1.51 (0.84-2.71)
Multivariate- adjusted*	1.00 (Reference)	0.81 (0.52-1.27)	1.15 (0.61-2.17)
Women	(n = 22)	(n = 34)	(n = 171)
Unadjusted	1.00 (Reference)	1.37 (0.24-7.82)	0.97 (0.21-4.45)
Multivariate- adjusted*	1.00 (Reference)	2.18 (0.37-12.69)	0.95 (0.22-3.93)

Data are presented as mean (standard error) or odds ratio (95% confidence interval).

*Adjusted for age, income status, education status, marital status, job status, amount of smoking, drinking status, BMI, physical activity, Hypertension, Diabetes mellitus, and Cerebrovascular disease.

Duration of smoking cessation among former smokers did not exhibit a significantly lower risk of depression in males. We did not evaluate the relationships between the duration of smoking cessation and depression in females because the number of former-smokers among females was small. Compared to those who quit smoking <5 years, the OR of those who quitted smoking ≥20 years was 0.92 (95% CI = 0.39–2.14) in males (Table 5).

**Table 5:** Odds ratio for depression according to duration of smoking cessation among former smokers in men

	**Duration of smoking cessation**			
	**< 5 years (n = 636)**	**5-9 years (n = 436)**	**10-19 years (n = 640)**	**≧ 20 years (n = 760)**
Men				
Unadjusted	1.00 (Reference)	0.92 (0.52-1.64)	1.48 (0.74-2.94)	1.83 (0.96-3.50)
Multivariate-adjusted*	1.00 (Reference)	0.71 (0.37-1.34)	1.32 (0.60-2.88)	0.92 (0.39-2.14)

Data are presented as mean (standard error) or odds ratio (95% confidence interval).

*Adjusted for age, income status, education status, marital status, job status, drinking status, pack-years, BMI, physical activity, Hypertension, Diabetes mellitus, and Cerebrovascular disease.

## Discussion

The results of this study demonstrated a stronger association between smoking status and depression in women than in men. However, the dose–response relationship for smoking and depression among current-smokers was only significant in females. No significant associations of the age of starting smoking and duration of smoking cessation with depression were observed among former-smokers.

In this study, the smoking rate in women was lower than that in men. The gender difference in smoking might be due to the effects of socio-cultural factors. Sex role norms and general expectations concerning gender-appropriate behavior have had a variety of effects on gender differences in smoking [21]. Traditional sex roles lead to social pressure against women smoking [21]. Additionally, differences in the demographic and socioeconomic characteristics of smokers and non-smokers have been reported [22-24]. The relationship between smoking and depression could be affected by these factors. However, it seems unlikely that these factors affected our results, as we adjusted for them in our analysis.

The CES-D is an adequate screening instrument for depressive disorder in the population [25-28]. We used a CES-D cutoff score of 21 as the definition of depression. Although the CES-D has low sensitivity and specificity for minor depression, it is excellent for use as a screening instrument for major depression, and the optimum cutoff point for the CES-D was found to be 21 [25]. Cho et al. also reported that a CES-D score of 21 most effectively detected a range of depressive symptoms during screening. They suggested that the higher cutoff point in Korea than in Western countries might be due to differences in the expression of affect, especially the suppression of positive affect, in cultures based on Confucian ethics [20].

Previous reports have proposed mechanisms for the association of smoking with depression. One pharmacological explanation for the influence of smoking on depression is that a reduction in monoamine oxidase B (MAO B) activity might synergize with nicotine to produce the diverse behavioral and epidemiological effects of smoking. The brains of smokers showed a 40% decrease in MAO B level relative to nonsmokers or former-smokers [29]. Another explanation is that vulnerability to depression is related to certain factors. Nicotine is known to affect the neurotransmitters involved in major depression [30]. Nicotine use might increase vulnerability to depression [31], and dysregulation of the dopaminergic system in an addictive state is a plausible mechanistic pathway to depressive vulnerability [32]. A further explanation is that smoking generates free radicals, causing lipid peroxidation, oxidation of proteins, and other tissue damage in smokers [33]. Depression has been associated with elevated reactive oxidative species such as anti-oxidative enzyme activities and lipid peroxidation [34].

Our data suggest that associations between smoking status and depression were more likely in females. Some studies reported a strong relationship between smoking and depression in females, rather than in males. Others reported a gender-specific effect, and that females showed a much greater smoking-depression relationship than males in both adolescents [14,35] and adults [15,16]. However, some studies have reported conflicting results. Nakata et al. reported that gender did not modify the effects of smoking on depression [36], and in some studies, an increased risk of depression was observed in male, but not female, smokers [37-39]. One possible explanation for this gender difference is that female smokers may have a lower threshold for depression than male smokers. McKee et al. reported that stressful life events were more strongly related to smoking in females than in males [40]. However, gender-specific effects in the smoking–depression relationship remain incompletely understood.

In this study, current-smokers had a higher risk of depression than never-smokers among both males and females. Although no statistically significant association was seen in males, former-smokers had a lower risk of depression than current-smokers. Wisebeck et al. reported that former-smokers carried a risk of depression that was significantly lower than that of current-smokers, but higher than in never-smokers [41]. They suggested that their results are in accordance with the hypothesis that smoking cessation was an effective way to reduce the risk of depression, and if so, depression should be less frequent in former-smokers who stopped smoking a long time ago compared to those who stopped recently [41]. However, in their study, the difference between the association of distant (first 3 years) smoking cessation and recent (within 1 year) smoking cessation with depression was not significant [41]. Mykletun et al. also reported no associations between depression and time since cessation in former-smokers [38]. They explained that although a trend toward a lower prevalence of depression with time since cessation was detected, it was not statistically significant [38]. Our results are similar; the risk of depression did not significantly decrease with increasing duration of smoking cessation in both males and females. We also evaluated the relationship between the age of starting smoking and depression, and no significant increased risk of depression was observed according to decreasing age of starting smoking. Some studies have reported that smoking during adolescence predicted depression later in life compared with never-smokers [14,42,43]. However, to our knowledge, no study had evaluated the relationship between the age of starting smoking and depression among current-smokers. We suggest that current smoking status is a more important factor for risk of depression.

If a physiologic link indeed exists between smoking and the subsequent development of depression, then it would be reasonable to expect that evidence of this link would became more pronounced as the number of cigarettes consumed increases. Klungsøyr et al. reported a dose–response relationship with an increasing risk for former-smokers and for increasing numbers of cigarettes smoked per day for current-smokers, and also for an increasing number of smoking-years [18]. Pasco et al. reported that compared with nonsmokers, the odds for major depressive disorder increases by 1.47-fold for females who smoked 11–20 cigarettes per day and more than doubled for those who smoked >20 cigarettes per day [44]. However, in their studies, dose–response relationships of smoking were evaluated by comparing them with never-smokers. Duncan and Reese reported very little evidence that smoking intensity was related to depression in smokers [45]. In their study, smoking an extra pack of cigarettes per month was associated with a statistically nonsignificant 0.02 increase in male CES-D scores and a 0.01 increase in female CES-D scores after controlling for smoking status [45]. In the present study, no dose–response relationship of smoking with depression was observed among current-smokers in males, but female heavy smokers had a higher risk of depression. We suggest that this difference might be related to a gender-specific effect of smoking on depression.

## Conclusions

Our data demonstrate that smoking is strongly associated with depression, particularly among females. However, a dose–response relationship was observed only between smoking and depression in females, and not in males, and duration of smoking cessation and age of starting smoking were not significantly associated with depression. Further large population-based prospective studies are needed to confirm the causal effects of smoking on depression. These findings suggest that depression prevention may need to be combined with smoking prevention and that different strategies in males and females may be necessary.

## Competing interests

The authors declare that they have no competing interests.

## Authors' contributions

WJY collected data, analyzed data and drafted the manuscript. MHS, SSK, SYR and JAR participated in the study design, data analysis and also reviewed the manuscript. All authors read and approved the final manuscript to be published.

## Pre-publication history

The pre-publication history for this paper can be accessed here:

http://www.biomedcentral.com/1471-2458/12/724/prepub
